# Long-read sequencing sheds light on key bacteria contributing to deadwood decomposition processes

**DOI:** 10.1186/s40793-024-00639-5

**Published:** 2024-12-03

**Authors:** Etienne Richy, Priscila Thiago Dobbler, Vojtěch Tláskal, Rubén López-Mondéjar, Petr Baldrian, Martina Kyselková

**Affiliations:** 1 Laboratory of Environmental Microbiology, Institute of Microbiology of the Czech Academy of Sciences, Vídeňská 1083, 14200 Prague 4, Czech Republic; 2 Institute of Soil Biology and Biogeochemistry, Biology Centre of the Czech Academy of Sciences, Na Sádkách 7, 37005 České Budějovice, Czech Republic; 3 Department of Soil and Water Conservation and Waste Management, CEBAS-CSIC, Campus Universitario de Espinardo, 30100 Murcia, Spain

**Keywords:** Deadwood microbiome, Biosynthetic gene cluster, Carbohydrate-active enzyme, Microbial decomposition, Metagenome-assembled genome, Metagenomics, Nitrogen fixation

## Abstract

**Background:**

Deadwood decomposition is an essential ecological process in forest ecosystems, playing a key role in nutrient cycling and carbon sequestration by enriching soils with organic matter. This process is driven by diverse microbial communities encompassing specialized functions in breaking down organic matter, but the specific roles of individual microorganisms in this process are still not fully understood.

**Results:**

Here, we characterized the deadwood microbiome in a natural mixed temperate forest in Central Europe using PacBio HiFi long-read sequencing and a genome-resolved transcriptomics approach in order to uncover key microbial contributors to wood decomposition. We obtained high quality assemblies, which allowed attribution of complex microbial functions such as nitrogen fixation to individual microbial taxa and enabled the recovery of metagenome-assembled genomes (MAGs) from both abundant and rare deadwood bacteria. We successfully assembled 69 MAGs (including 14 high-quality and 7 single-contig genomes) from 4 samples, representing most of the abundant bacterial phyla in deadwood. The MAGs exhibited a rich diversity of carbohydrate-active enzymes (CAZymes), with Myxococcota encoding the highest number of CAZymes and the full complement of enzymes required for cellulose decomposition. For the first time we observed active nitrogen fixation by Steroidobacteraceae, as well as hemicellulose degradation and chitin recycling by Patescibacteria. Furthermore, PacBio HiFi sequencing identified over 1000 biosynthetic gene clusters, highlighting a vast potential for secondary metabolite production in deadwood, particularly in Pseudomonadota and Myxococcota.

**Conclusions:**

PacBio HiFi long-read sequencing offers comprehensive insights into deadwood decomposition processes by advancing the identification of functional features involving multiple genes. It represents a robust tool for unraveling novel microbial genomes in complex ecosystems and allows the identification of key microorganisms contributing to deadwood decomposition.

**Supplementary Information:**

The online version contains supplementary material available at 10.1186/s40793-024-00639-5.

## Introduction

Deadwood constitutes a fundamental component of natural forest ecosystems, providing a variety of niches for microorganisms and representing a hotspot of biodiversity [1]. Decomposing deadwood releases carbon dioxide into the atmosphere, but also enriches the soil with nutrients and recalcitrant organic matter (OM), thereby promoting nutrient cycling and enhancing long-term carbon storage in forest soils [2]. This decomposition process is primarily driven by wood-decaying fungi, such as basidiomycetes, and bacteria, including Alphaproteobacteria, Gammaproteobacteria, Acidobacteriota, and Actinomycetota [3, 4]. Fungi initiate the decomposition of fresh deadwood by depolymerizing the wood biopolymers including lignin and cellulose, thereby enabling subsequent colonization of wood matrix and its utilization [5, 6]. Nitrogen-fixing bacteria also play a crucial role in early stages of decomposition by mitigating the high carbon-to-nitrogen ratio typical of fresh deadwood, which supports subsequent microbial activity [7]. As decomposition progresses, fungi and bacteria collaboratively degrade plant cell wall components like cellulose and hemicellulose, using a wide range of extracellular hydrolytic enzymes.

Second-generation (short-read) sequencing has significantly advanced our understanding of the taxonomic composition and functional potential of deadwood microbial communities [4, 8–11]. However, this approach often results in incomplete and fragmented metagenome-assembled genomes (MAGs) due to limited assembly capabilities, hampering the recovery of complex genomic features such as complete operons, large genomic islands, or repetitive sequences. These limitations hinder the ability to fully explore the functional potential of the microbial community and achieve accurate taxonomic classification [12]. In contrast, third-generation (long-read) sequencing technologies such as PacBio HiFi sequencing, produce highly accurate long reads, yielding more complete and less contaminated MAGs [13, 14]. The increased read length enhances the resolution of intricate genomic structures, providing a deeper insight into the ecological roles of microorganisms.

In this study, we aimed to characterize the key microorganisms involved in deadwood decomposition in a natural mixed temperate forest from Central Europe, using PacBio HiFi sequencing in combination with a genome-resolved transcriptomics approach. Using deadwood samples previously analyzed through bacterial genomics, metagenomics and metatranscriptomics with second generation high-throughput sequencing [9–11, 15], we also aimed to evaluate the added value of PacBio HiFi sequencing over short-read sequencing for characterizing the deadwood microbiome. We anticipated that the long-read lengths and high accuracy of PacBio HiFi sequencing would yield more complete and less contaminated MAGs from the most abundant taxa and provide better resolution of operons and gene clusters encoding specific metabolic functions.

## Material and methods

### Sample description

**Fig. 1:**
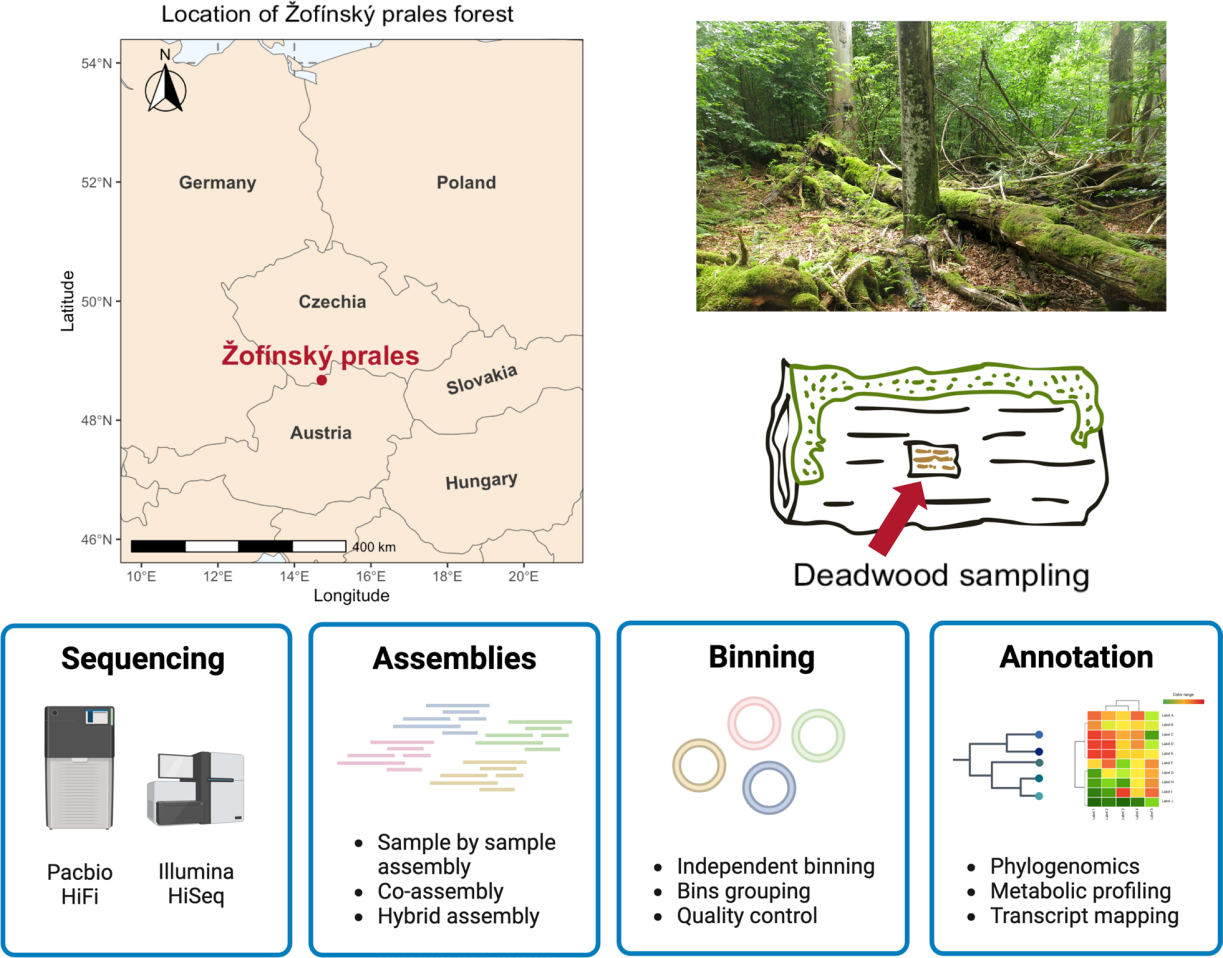
Sampling strategy and metagenomic workflow. Deadwood samples (n = 4) were collected from the Žofínský Prales National Nature Reserve, Czech Republic. For each sample, *Fagus sylvatica* trunks were drilled vertically from the middle of the top surface at five equidistant locations, and sawdust from the inside of the trunks was collected and pooled. The four samples were sequenced using Pacbio HiFi and Illumina HiSeq. Sample-by-sample assembly and co-assembly was performed using short and long reads, as well as hybrid assembly. Assemblies were binned independently and bins were pooled, with the exception of the hybrid assembly, which gave unsatisfactory results. After quality control (completeness and contamination, dereplication and mis-binning detection), the phylogeny and metabolic capacities of the metagenomes-assembled genomes (MAGs) were analyzed. Transcripts were mapped to MAGs to validate the functionality of specific metabolism. The figure illustrating the metagenomic workflow was created with Biorender.com

Deadwood samples were collected in the core zone of the Žofínský prales National Nature Reserve in the Czech Republic (48°39′57″N, 14°42′24″E), a forest protected since 1838. Detailed site descriptions and deadwood sampling methods are provided in Tláskal et al. [9]. A map generated with the r package *sf* [16] representing the location of the sampling site is provided in Fig. 1. Four samples from *Fagus sylvatica* trunks representing class 2 (4–7 years of decomposition; sample 6—BioSample accession SAMN13925154 and sample 7—SAMN13925155) and class 4 (20–41 years of decomposition; sample 57—SAMN13925167 and sample 84—SAMN13925168) were analyzed. Samples were stored at − 80 °C prior DNA isolation. Short-read sequencing using Illumina HiSeq platform was previously performed on these samples [9, 10] and the short-read data (accessible via the BioProject accession number PRJNA603240) were used in the present study.

### DNA isolation for PacBio HiFi sequencing

High molecular weight DNA was isolated from the four deadwood samples using a phenol/chloroform/isoamyl alcohol extraction method according to Sagova-Mareckova et al. [17] with several modifications; notably we replaced the bead-beating step by vortexing in order to prevent excessive DNA shearing. Samples were first thoroughly ground in a sterile mortar with liquid nitrogen using a rough pestle. Sample aliquots (~ 250 mg) were added to 2 mL tubes with a screw lid and mixed with 600 µL extraction buffer (50 mM Na-phosphate buffer pH 8, 50 mM NaCl, 500 mM Tris–HCl pH 8, and 5% sodium dodecyl sulfate) and 300 µL phenol (pH 8)/chloroform/isoamyl alcohol (25:24:1). The tubes were vortexed for 1 min at 4 °C, using the Vortex-Genie (Scientific Industries, Bohemia, NY) set at the maximum speed. The homogenized samples were centrifuged at 10,000 g for 3 min and supernatant was transferred to a clean tube. One volume of phenol/chloroform/isoamyl alcohol (25:24:1) was added and mixed with the supernatant by gentle tube inverting for 1 min. After centrifugation at 6000 g for 5 min, the supernatant was transferred to a clean tube, mixed (gentle tube inverting for 1 min) with 1 volume of chloroform/isoamyl alcohol (24:1), and centrifuged (6000 g, 5 min). The supernatant was transferred to a clean tube and mixed with NaCl (to a final concentration of 1.5 M) and CTAB (to a final concentration of 1%), and incubated at 65 °C for 35 min. The incubated solution was cooled at 4 °C for 5 min and then mixed with an equal volume of chloroform/isoamyl alcohol (24:1), and centrifuged at 3400 g for 20 min. The supernatant was mixed with 0.6 volume of isopropanol and 0.1 volume of 3 M sodium acetate in a clean tube and incubated at 4 °C for 45 min to precipitate the DNA. After centrifugation at 10,000 g for 20 min, the supernatant was removed and the DNA pellet was washed with 200 µL cold 70% ethanol, air-dried and resuspended in 30 µL 10 mM Tris buffer pH 8. Presence of high molecular weight DNA was verified with DNA electrophoresis in 0.8% agarose gel and DNA concentration was measured with Qubit 2.0 Fluorometer (Invitrogen, Carlsbad, CA). Three to six DNA aliquots per sample were pooled to obtain the minimal required DNA quantity of 5 µg.

### Sequencing and primary analyses of the metagenomes

The library preparation and PacBio HiFi sequencing on the Sequel II Instrument were performed at Brigham Young University Sequencing Centre, Utah. Samples 6 and 7 were pooled equimolarly during library preparation and sequenced together on one 8 M SMRT Cell using 30-h movie. Samples 57 and 84 were sequenced separately on one 8 M SMRT Cell using 30-h movie. PacBio’s movie subreads files were processed with CCS (https://github.com/PacificBiosciences/pbbioconda) for consensus Hi-Fi reads of > 99% accuracy. After removing reads of less than 1000 bp, a total of 866,007 sequences with a total length of 16.1 Gb were obtained from the 4 samples. Taxonomic profiling of the raw PacBio HiFi reads was performed using DIAMOND + MEGAN-LR workflow. Reads were aligned with DIAMOND v2.1.8 [18] blastx against the NCBI nr database (version 09/2022) using the following parameter ‘-range-culling -top 5- F 5000’ for long-read mode, followed by ‘meganization’ to ensure the LCA (last common ancestor) assignation for each read. For this, the MEGAN6 Community Edition [19] ‘daa2rma’ function was used with the following parameter ‘-longReads-lcaAlgorithm longReads-ram readCount’.

See Supplementary Material for details on primary analyses of the short-read metagenomes and metatranscriptomes.

### Sequence assembly and taxonomic profiling

Sequences generated by PacBio HiFi sequencing were co-assembled using Hifiasm-meta r058 [20] with default parameters, and metaFlye v2.9.2 [21] with –pacbio-hifi and –meta setting [22]. Hifiasm-meta produced a total of 16,446 contigs with an N50 of 80,906bp, and metaFlye produced a total of 6589 contigs with a N50 of 90,042bp (Table S1). As a result, only Hifiasm-meta co-assembly was further analyzed. This assembler was also chosen for sample-by-sample assemblies. See Supplementary Material and Table S2 for details on the assembly of short-read and hybrid assemblies. Prior taxonomic classification, gene prediction was conducted using FragGeneScan v1.31 [23] on the assembled contigs from both co-assemblies and individual sample assemblies. The predicted genes were then aligned to the NCBI nr database (version 09/2022) using the DIAMOND blastp tool. Taxonomy was assigned based on the best-hit criteria (lowest e-value, highest identity, and highest bit-score), with a minimum e-value threshold of 10E-5.

### MAG recovery and taxonomic identification

Bins were generated using Pacbio HiFi sample-by-sample assembly and co-assembly in parallel. We employed three binning tools (MetaBAT v2.12.1, MaxBin v2.2.6, and CONCOCT v1.0.0) with default settings within metaWRAP v1.3.2 [24]. The resulting bins were next combined and refined using the Bin_refinement module of metaWRAP with the parameters ‘-c 50—× 10’ to obtain at least medium-quality (i.e., completeness > 50%, contamination < 10%) draft MAGs. CheckM v1.0.12 [25] lineage workflow in metaWRAP was used to assess the quality (completeness and contamination) of the MAGs and seqkit v2.3.0 [26] was used to obtain basic MAG statistics. MAGs were dereplicated using dRep v3.4.0 [27]. First, genomes were grouped at ~ 90% ANI based on k-mer content using Mash [28] (primary clustering). These groups were then refined by aligning genomes within each primary cluster at 95% ANI with ANImf [29] (secondary clustering). Only non-redundant genomes with the highest completeness and the lowest contamination were kept for further analysis. We used GTDB-Tk v2.1.0 [30] to assign taxonomy to these MAGs based on the taxonomy R207_v2 from the Genome Taxonomy Database (GTDB). We further re-estimated the completeness and the contamination of the Patescibacteria MAGs using the recently developed Checkm2 v0.1.3 [31], providing better estimates for this phylum. We screened for the presence of ribosomal RNAs (i.e., 16S, 23S and/or 5S rRNA) in MAGs using barrnap v0.9 (https://github.com/tseemann/barrnap). The same procedure was used to generate the MAGs from Illumina HiSeq sequencing, but these MAGs were used for statistical comparison only (Figure S1, Supplementary Material).

Functional annotation of predicted ORFs was done with DRAM v1.4.0 [32], which relied on Pfam, KOfam, UniProt, dbCAN, and MEROPS. We estimated the relative abundances of the MAGs across the four metagenome datasets using the CoverM v0.6.1 tool [33], by assessing the coverage of mapped long-read in ‘genome’ mode. To obtain relative abundance, the average genome coverage was divided by the average total coverage of all genomes, then normalized by the ratio of mapped reads to total reads. A final contamination assessment and chimerism check on the final set of MAGs was done using GUNC v1.0.5 [34].

### Integrating MAGs and metabarcoding data

The prokaryotic diversity of the samples was estimated using 16S rRNA amplicon sequencing data and compared with the 16S rRNA profiles of MAGs. First, Illumina MiSeq (2 × 250 bases) metabarcoding data for these samples were obtained from Tláskal et al., [10]. Amplicon sequencing data were then processed using the SEED v2.1.05 pipeline [35], following the methods of Richy et al. [36]. Paired-end reads were joined using fastq-join, and sequences with ambiguous bases or a mean quality score below 30 were excluded. Chimeric sequences were identified and removed using USEARCH v11.0.667 [37], followed by clustering with UPARSE within USEARCH [38] at 97% of similarity. The most abundant sequences were chosen as representatives for each Operational Taxonomic Unit (OTU), singletons were discarded, and OTU tables were rarefied to a common sampling depth of 9164 reads per sample using the rarefy_even_depth() function in the *phyloseq* R package [39]. Species-level matches were determined by BLASTn against Silva v138.1 [40]. Hill numbers were used to calculate diversity indexes (observed richness = H0 and exponential of Shannon = eH’) using the DivPart() function in the *entropart* R package [41]. Finally, the 16S rRNA sequences of the MAGs were aligned and mapped to the 16S rRNA amplicon sequences using blastn (maximum e-value of 1 × 10^–5^) and bowtie2 v2.4.1 [42], respectively. Alignment and mapping results were consistent for 45 out of the 48 MAGs possessing a 16S rRNA copy in their genome (Table S3), and agreed with the MAG GTDB taxonomy.

### Functional characterization of the deadwood microbial communities

Relevant functions in wood decomposition were screened [9]. All these analyses were performed on short reads, long reads and MAGs. EukRep v0.6.7 [43] were used to predict contigs of eukaryotic origin. Metatranscriptomes were used to validate the expression of specific metabolisms.

### Characterization of carbohydrate-active enzymes (CAZymes)

Genes involved in the decomposition of the easily degradable carbohydrates (arabinogalactanases, xylanases/xyloglucanases, mannanases, cellobiases and xylobiases), complex plant cell wall biopolymers (endoglucanase, cellobiohydrolase, exoglucanase), microbial biomass (peptidoglycansases, beta-glucanases, chitinases) and reserve compounds (alphaglucanases) were predicted using FragGeneScan v1.31 [23], and annotated using run_dbcan.py (v2) program [44]. Predicted prokaryotic and eukaryotic genes were compared to the dbCAN database V9 using HMMER 3.0 [45]. See Table S4 for the details of the CAZymes screened for each activity. To validate Myxococcota cellulose decomposition activity, we mapped transcripts from sample 7 to Myxococcota MAGs (both Myxococcota genomes were assembled from this sample) using minimap2 v2.24 [46] with the ‘-x sr’ setting. Transcript counts were summed using dirseq v0.4.3 [47] based on gene coordination from the gene prediction.

### Nitrogen metabolism investigation

Predicted genes from long-read and short-read co-assemblies were first screened with HMMER v3.3.2 against the KOfam database (accessed in June 2022) using the predefined adaptive thresholds [48]. After detecting the metabolic pathways involved in the nitrogen cycle found in a single contig, we further analyzed the contigs in which regions associated with N_2_ fixation were found (i.e. *nif* genes + *fix* genes + Isc system genes or Suf system genes). We performed gene prediction using GeneMarkS v1.14 [49] with default setting, and the annotation using eggnog-mapper v2.1.11 [50] and MMseqs after translating input CDS to proteins. The phylogenetic relationships between the *nifH* sequences were analyzed using neighbor-joining trees generated using the maximum likelihood algorithm with 1000 bootstrap iterations. First, multiple sequence alignment was generated using mafft v7.490 [51] with the option ‘–maxiterate 1000’ and ‘–localpair’, then sequences were trimmed using BMGE v.2.0 [52] software and the BLOSUM30 matrix. Finally, phylogenetic tree was computed using iqtree v2.2.0.3 [53] and the LG + C20 model and visualized using FigTree v1.4.4 (http://tree.bio.ed.ac.uk/software/figtree/). Additional analyses on the nitrogen fixation potential in Steroidobacteraceae MAG are described in the Supplementary Material.

### Biosynthetic gene clusters

AntiSMASH v6.1.1 [54] was used to predict secondary metabolite clusters. BGCs were predicted with ‘–hmmdetection-strictness strict’ and ‘–asf’ settings. The options ‘–cb-general’, ‘–cb-subclusters’ and ‘–cb-knownclusters’ were added in order to compare identified clusters against antiSMASH-predicted clusters database, known subclusters responsible for synthesizing precursors and known gene clusters from the MIBiG database [55] respectively. To cluster the BGCs by similarity, we used BiG-SCAPE v1.1.5 [56] with the ‘–mibig’ and ‘–cutoffs 0.6’ flags to group the BGC detected and the MIBiG database v3.1 entries at 60% of similarity.

## Results

### PacBio HiFi sequencing yields high-quality assemblies

The PacBio HiFi sequencing approach produced an average of 216,502 sequences per sample, with an average read length of 18,340 bp (Table S5). Eighty-five percent of the raw reads were assigned to Prokaryotes (mostly Bacteria) and 13% to Eukaryotes (Table S6). The co-assembly of PacBio HiFi reads yielded 16,446 contigs, with an N50 of 66,188 bp, including 16,439 contigs > 10 kb and 9696 contigs > 50 kb (Table S1). The largest contig reached 5.8 Mb. On average, 41% of the long reads mapped to the assembled contigs (Table S7). Most contigs were assigned to Bacteria, while 10% were assigned to Eukaryotes (Table S6). See Tables S2 and Tables S6 for details of sample-by-sample assembly.

### MAGs from both abundant and rare bacterial taxa recovered with PacBio HiFi data

Binning based on PacBio HiFi assemblies generated a total of 69 unique bacterial MAGs composed of 26 ± 21 contigs with an average length of 148,129 ± 386,395 bp (Table S8). Among these, 14 were High-quality draft MAGs (i.e., completeness > 90% and contamination < 5%, presence of the 23S, 16S, and 5S rRNA genes and at least 18 tRNAs) and 55 were Medium-quality draft MAGs (completeness > 50%, contamination < 10%, Figure S1). Twenty-two MAGs were composed of less than 10 contigs, among which 7 were composed of a single contig and considered nearly finished MAGs (Table S8).

**Fig. 2:**
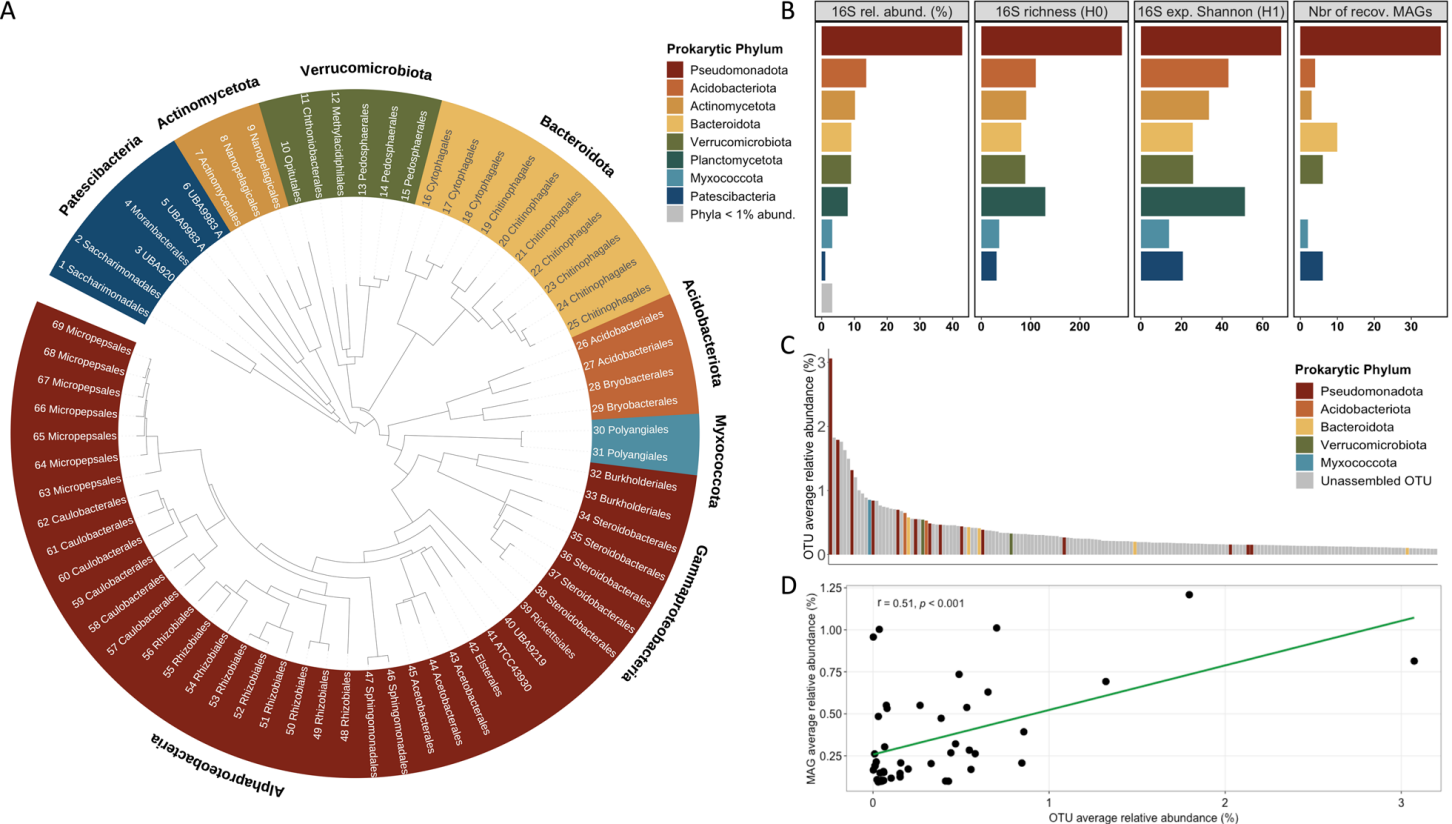
Integrating de novo genome assembly and metabarcoding. **A** Maximum likelihood phylogenomic tree, classifying MAGs based on their position in the reference tree, relative evolutionary divergence, and average nucleotide identity (ANI) compared to reference genomes. The tree was generated using GTDB-tk based on the concatenated phylogeny of 120 bacterial single-copy marker genes and includes 69 unique bacterial genomes (MAGs) with completeness > 50% and contamination < 10%. **B** Relative abundance of sequences (%), phylum richness (H0) and exponential of Shannon (H1) index estimated from 16S rRNA amplicon metabarcoding and averaged across the four samples, as well as number of recovered MAGs per bacterial phyla. **C** Barplot displaying the averaged OTU relative abundance across all four samples. Only OTUs with an average relative abundance > 0.1% (top 172) are shown and ranked in descending order. The bar color corresponds to the phylum when a corresponding MAG was assembled. **D** Correlation between MAG relative abundance and sequence relative abundance of corresponding OTUs

These MAGs were assigned to Pseudomonadota (38 genomes), Bacteroidota (10), Verrucomicrobiota (6), Patescibacteria (6), Acidobacteriota (4), Actinomycetota (3), and Myxococcota (2) (Fig. 2A). Genomes from the most abundant bacterial phyla identified through metabarcoding were successfully binned (Fig. 2B), and the number of recovered MAGs was strongly correlated with the OTU richness of these phyla (Pearson correlation coefficient, r = 0.84, p = 0.008). However, no genomes from the Planctomycetota phylum were recovered, although their contigs were successfully assembled (Figure S2). This likely reflects the unique community structure of this clade, which had relatively low 16S rRNA abundance (ranking as the sixth most abundant phylum) but was the second most diverse phylum after Pseudomonadota (Fig. 2B).

Out of the 48 MAGs containing a copy of the 16S rRNA gene, 44 MAGs were successfully linked to a unique OTU recovered from metabarcoding data. Among these, 24 were associated to OTUs exhibiting a relative abundance greater than 0.1% (i.e., among the 172 OTUs, Fig. 2C), including five MAGs originating from the 12 most abundant OTUs assigned to Alphaproteobacteria (3) and Myxoccocota (2). Additionally, 20 MAGs corresponded to OTUs with a relative abundance below 0.1%, including two MAGs assigned to Patescibacteria and two to Alphaproteobacteria. The proportional relationship between MAG recovery and OTU relative abundance (Fig. 2D) confirmed that genomes from both abundant and rare taxa were successfully recovered.

### Functional profiling reveals key bacteria involved in OM decomposition

**Fig. 3:**
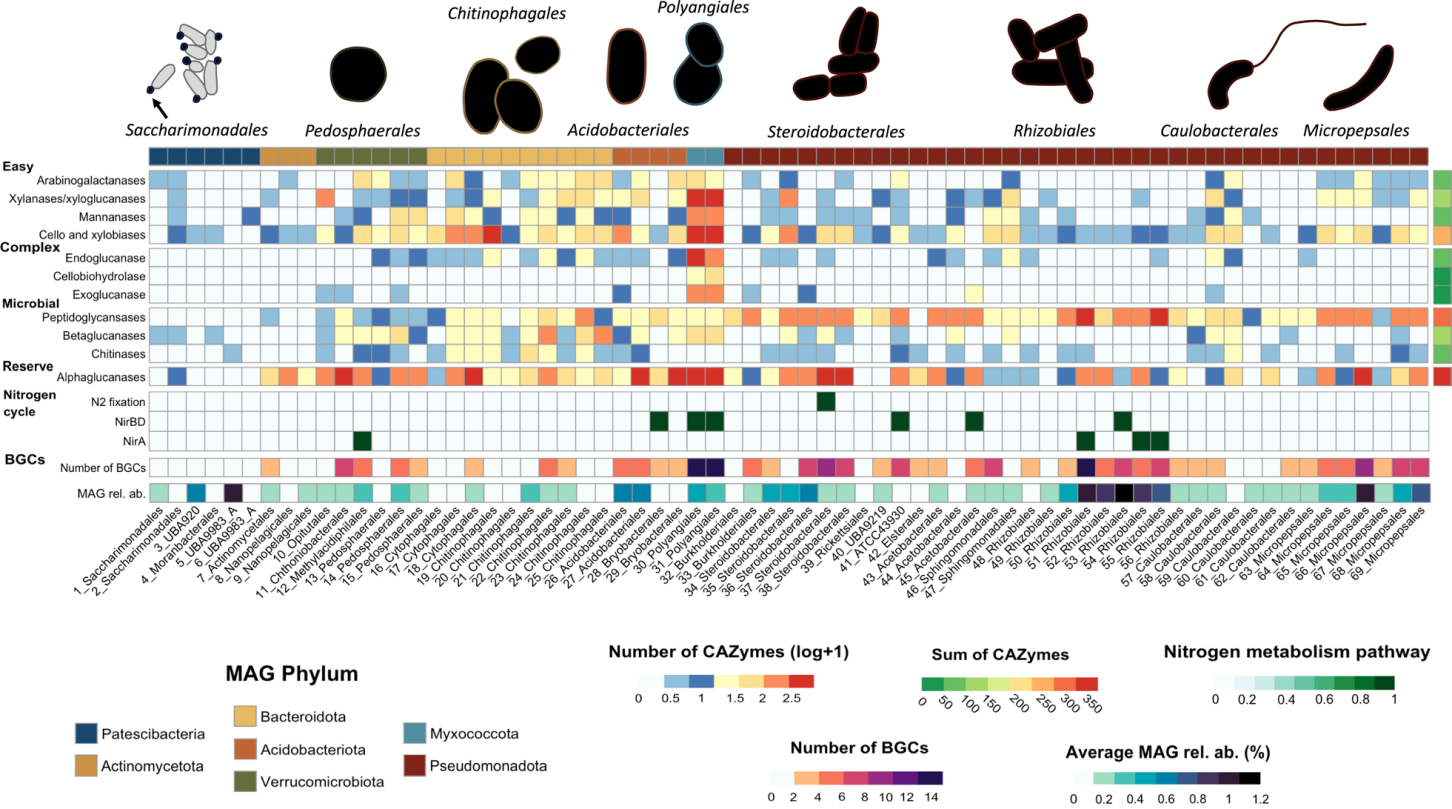
Functional profiles of deadwood-associated MAGs. Heatmap representing the metabolic capacities involved in the decomposition of the easily decomposable carbohydrates (arabinogalactanases, xylanases/xyloglucanases, mannanases, cellobiases and xylobiases) and complex plant cell wall polymers (endoglucanases, cellobiohydrolases, exoglucanases), microbial biomass (peptidoglycansases, betaglucanases, chitinases), and reserve compounds (alphaglucanases), nitrogen fixation, dissimilatory nitrate reduction (nitrite to ammonia only) and assimilatory nitrate reduction (nitrite to ammonia only), and the number of biosynthetic gene clusters found in the 69 MAGs. The top row represents the phylum to which the MAG was assigned. The number of CAZymes were log transformed + 1 to improve the visualization, and the total counts of CAZymes were summed on the right last column per activity or by class of enzyme. The bottom row represents the average relative abundance of the MAG. The shape of the bacteria was drawn based on the closest characterized clades of each MAG lineages

The abundance of CAZymes was comparable across short reads, long reads, and MAGs, with alphaglucanases, peptidoglycanases, cellobiases, and xylobiases being the most common, while exoglucanases and cellobiohydrolases were relatively rare (Figure S3). MAGs assigned to Myxococcota (Polyangiaceae family) encoded the highest number of CAZymes involved in the degradation of both easily decomposable and recalcitrant biopolymers (Fig. 3). These MAGs encoded the full complement of enzymes required for cellulose decomposition, including endoglucanases, exoglucanases, and cellobiohydrolases. Importantly, while endoglucanases and exoglucanases were transcribed by other MAGs, transcript mapping revealed that cellobiohydrolases were exclusively transcribed by Polyangiaceae (Table S9). Bacteroidota also exhibited a high number of CAZymes targeting easily degradable carbohydrates, as well as microbial biomass. In contrast, Acidobacteriota and Verrucomicrobiota encoded fewer enzymes for microbial biomass recycling but many for easily degradable carbohydrates and reserve compounds. Pseudomonadota exhibited functional versatility, encoding numerous CAZymes for microbial biomass degradation, though the distribution of enzymes for other substrates was inconsistent.

The number of CAZymes found in MAGs was proportional to their genome size (r = 0.76, p-value < 0.001). Consequently, the phyla encoding the smallest number of CAZymes were Actinomycetota and Patescibacteria. Actinomycetota assembled in deadwood (Nanopelagicaceae and Microbacteriaceae families) encoded mainly CAZymes targeting reserve compounds and easily degradable carbohydrates, and lacked the metabolic capacity to degrade complex plant cell wall biopolymers and microbial biomass (Fig. 3). Patescibacteria exhibited only few enzymes involved in decomposition of easily degradable carbohydrates, targeting microbial biomass and reserve compounds. However, transcript mapping confirmed expression of CAZymes by Patescibacteria, exceeding the transcription of Acidobacteriota and Pseudomonadota for mannanase and betaglucanases activity (Table S9). In addition, while overall chitinase expression was low among the MAGs recovered (mean coverage = 3.0), Patescibacteria exhibited the second-highest chitinase expression after Bacteroidota (6.6 and 7.3 respectively).

### New nitrogen-fixing bacteria identified in deadwood

While no complete nitrogen cycle pathways were detected on contigs from short-read sequencing, PacBio HiFi contigs and MAGs revealed the presence of dissimilatory and assimilatory nitrate reduction pathways, along with nitrogen fixation (Table S10). The *nirB* and *nirD* genes, which convert nitrite to ammonia in the dissimilatory nitrate reduction pathway, were found in Pseudomonadota (including three Alphaproteobacteria MAGs), Myxococcota (two Polyangiales), and Acidobacteriota (Bryobacterales) (Fig. 3). The *nirA* gene, responsible for the conversion of nitrite to ammonia in the assimilatory nitrate reduction pathway, was identified in Verrucomicrobiota (Methylacidiphilales) and Alphaproteobacteria (three Rhizobiales). Nitrogenase genes (*nifH*, *nifD*, *nifK*), which catalyze the biological reduction of dinitrogen to ammonia, were exclusively present in the MAG 36_Steroidobacterales (a gammaproteobacterium assigned to the family Steroidobacteraceae).

**Fig. 4:**
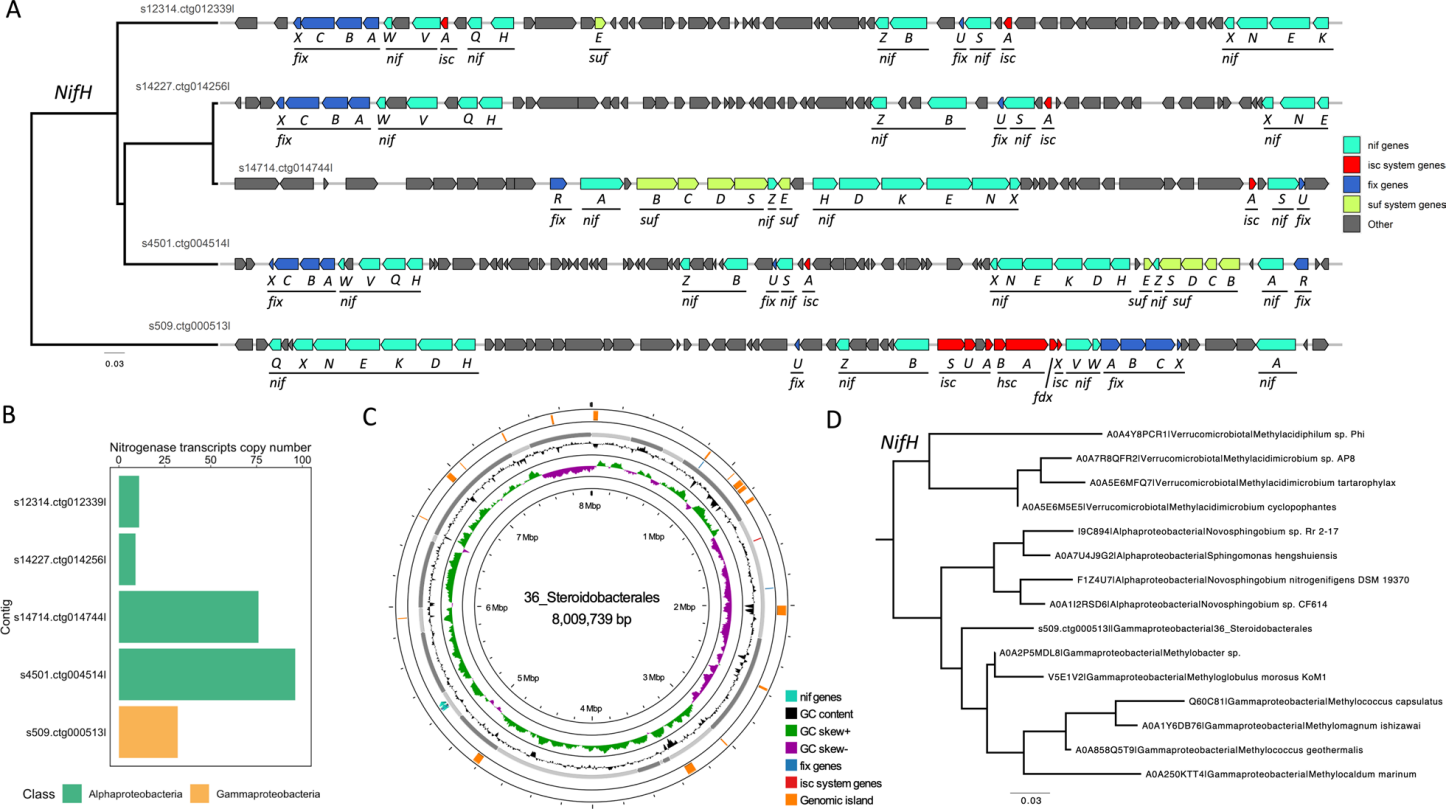
Characterization of the genomic regions associated with N_2_ fixation. **A** Gene organization of the five genomic regions associated with N_2_ fixation assembled from PacBio HiFi assembly. Clustering is based on the phylogenetic relationships of the corresponding NifH sequences. Only regions associated with N_2_ fixation were plotted. The genes are colored by operons and systems: in cyan the *nif* genes, in blue the *fix* genes, in red the Isc system genes and in green the Suf system genes. **B** Numbers of transcripts mapping to the nitrogenase genes (*nifHDK*) found in the five contigs (from A), belonging either to Alphaproteobacteria (green) or Gammaproteobacteria (yellow). The contig s509.ctg000513l corresponds to the contig found in the MAG 36_Steroidobacterales. **C** Circular plot illustrating genomic islands of the MAG 36_Steroidobacterales computed with Islandviewer4. The exact location of the nitrogen fixation contig s509.ctg000513l is between 5,118,742 and 5,335,241 bp, where no genomic island was detected. **D** Phylogenetic analysis placing the *nifH* gene found in the MAG 36_Steroidobacterales within Gammaproteobacteria. Neighbor-joining trees were generated using the maximum likelihood algorithm with 1000 bootstrap iterations and rooted using minimal ancestor deviation. First, the NifH amino acid sequences from the InterPro database (accessed on August 1st, 2023) clustered at 97% of similarity were included, followed by sub-selection of the closest related taxa

The discovery of nitrogenase genes within the Steroidobacteraceae family was unexpected, but supported by subsequent analyses which indicated accurate binning, no recent N_2_-fixation gene transfer, and active nitrogen fixation by this MAG (Fig. 4 and Supplementary Material). To further characterize the nitrogen-fixing capabilities of the MAG 36_Steroidobacterales, we compared its genomic regions associated with N_2_ fixation to other contigs generated by PacBio HiFi co-assembly (Fig. 4). We identified four additional contigs containing essential nitrogen fixation genes, all assigned to Alphaproteobacteria (Table S11). The minimum set of genes required for nitrogen fixation, specifically *nifHDK* and *nifENB*, were present. Additionally, the *fixABCRUX* genes were identified, preferred over the Rnf complex for producing reduced ferredoxin/flavodoxin. Although 36_Steroidobacterales lacked *nifS*, it encoded the complete *Isc* system (*iscR*, *iscS*, *iscU*, *iscA*, *hscB*, *hscA*, *fdx*, and *iscX*), essential for the maturation of [Fe-S] proteins. In contrast, the Alphaproteobacteria contigs lacked the *Isc* system but contained the *Suf* system (*sufB*, *sufC*, *sufD*, *sufS*, and *sufE*) instead.

### Deadwood bacterial communities exhibit a wide range of biosynthetic gene clusters

A total of 1089 putative BGCs were identified in the PacBio HiFi long-read co-assembly, presenting a stark contrast to the three BGCs found in the short-read co-assembly (Figure S4). A comparison with the MIBig database highlighted the novelty of these BGCs, with a staggering 98% showing less than 50% shared genes with MIBig entries. The primary BGC types identified in the long-read assembly were also found in MAGs, enabling the association of biosynthetic potential with taxonomic groups (Table S12).

**Fig. 5:**
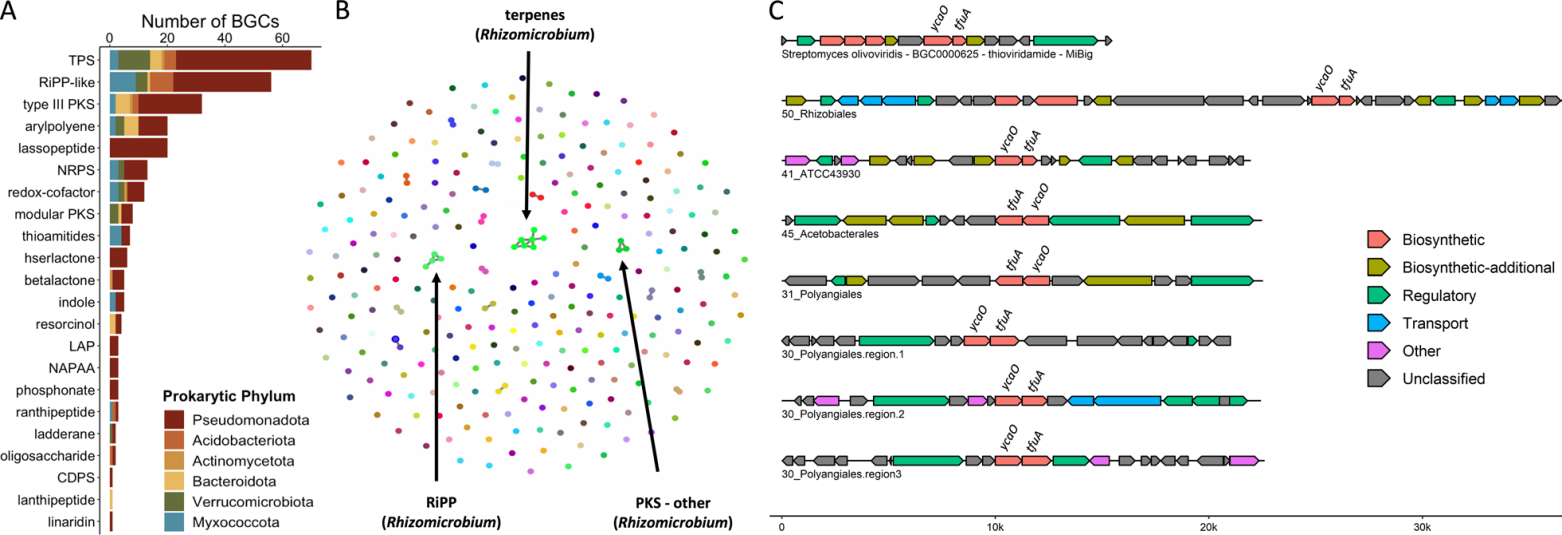
Biosynthetic gene cluster diversity in MAGs. **A** Barplot summarizing the number of BGCs identified in MAGs derived from long-read co-assembly. BGCs are categorized by type and ranked in descending order. Colors indicate the number of BGCs per phylum. TPS, terpenes; RiPP, ribosomally synthesised and post-translationally modified peptides; PKS, polyketide synthase product; NRPS, non-ribosomal peptide synthetase product; LAP, linear azol(in)e-containing peptides; NAPAA, non-alpha poly-amino acids like e-polylysin; CDPS, tRNA-dependent cyclodipeptide synthase product. **B** Similarity network of 279 BGCs found in 69 MAGs. BGCs belonging to the same gene cluster family are linked. Gene cluster families with more than 2 members are labeled with the corresponding product type and taxonomic classification of the MAGs in which they occurred (in parenthesis, all BGCs within one gene cluster family always originated from the same genus). **C** Organization of BGCs encoding thioamitides. Seven thioamitide BGCs found in 5 MAGs (each belonging to a unique gene cluster family) are displayed together with thioviridamide BGC from *Streptomyces olivovoridis* as a reference. The core biosynthetic genes *ycaO* and *tfuA* are labeled

Pseudomonadota, particularly MAGs from Rhizomicrobium, Rhizobiales (Alphaproteobacteria), and Steroidibacteriaceae (Gammaproteobacteria), carried the majority of BGCs, exceeding 10 in some instances (Fig. 5A). Pseudomonadotal BGCs presented a wide diversity, encompassing various types of compounds such as thioamitides, lasso peptides, ranthipeptides, azol(in)e-containing linear peptides, linaridines, or ladderanes, each holding bioprospecting potential. The two Myxococcota MAGs displayed the highest numbers of BGCs (14 and 15), encoding a wide array of compounds, including thioamitides and ranthipeptides. Verrucomicrobiota, Acidobacteriota, Bacteroidota, and Actinomycetota MAGs harbored 0–8 BGCs, with terpene, RiPP, polyketide, NRPS, and arylpolyene BGCs being the most prevalent. Group-specific BGCs, such as flexirubins in Chitinophagaceae (Bacteroidota) MAGs, were also identified. Patescibacteria MAGs did not contain any BGCs. BiG-SCAPE analysis revealed that the 271 BGCs identified in MAGs were categorized into 254 unique gene cluster families (Fig. 5B). The abundance of singletons underscored the vast diversity of BGCs in deadwood MAGs. For instance, seven BGCs encoding thioamitides each belonged to a distinct gene cluster family (Fig. 5C). BGCs belonging to one cluster family always belonged to taxonomically related MAGs, indicating that BGCs in deadwood bacteria are phylogenetically conserved.

## Discussion

PacBio HiFi metagenomic sequencing has demonstrated outstanding efficiency for reconstructing genomes from deadwood microbial communities, including Myxococcota, which could not be previously recovered through cultivation or short-read metagenomics from these samples [10, 15]. This technology enabled the assembly of both abundant and rare bacterial genomes, allowing better characterization of the deadwood bacterial communities. A total of 69 bacterial genomes were generated from 4 deadwood samples, including 67 newly reconstructed MAGs, 14 high-quality and 7 composed of a single contig. While PacBio HiFi generated a comparable amount of data to Illumina sequencing (an average of 4.0 Gb versus 4.9 Gb per sample respectively, Table S5), it outperformed Illumina in binning (69 versus 11 MAGs), yielding less fragmented, less contaminated, and more complete MAGs. In comparison to related studies, our findings overtake those of Tláskal et al. [10], who assembled 58 MAGs from 25 short-read metagenomes of deadwood from the same forest. Our investigation extended to the eight most abundant deadwood bacterial phyla, showing successful genome assembly for all phyla except Planctomycetota. Our results suggest that while genome size and relative abundance do not appear to be the ultimate limiting factors for successful binning, the inherent complexity of Planctomycetota, characterized by high alpha diversity, challenges their genome recovery.

Employing PacBio HiFi sequencing, we underscored the important role of Myxococcota in deadwood decomposition. While Myxococcota have been previously assembled from various ecosystems, including aquatic, terrestrial, host-associated, and built environments [57], our study marks the first assembly of Myxococcota genomes from deadwood samples. The successful recovery of these genomes using long-read sequencing—where previous short-read sequencing efforts failed [10]—is likely due to the large size of Myxococcota genomes (> 8 Mbp, according to the GTDB database r202) and the frequent presence of complex genomic features, such as insertion sequences, prophages, genomic islands, and hypervariable regions with high single nucleotide polymorphism density [58]. These features are challenging to assemble into MAGs with short-read data. Long-read sequencing, with its capacity to span repetitive and variable regions, is thus more effective at capturing the complete and contiguous genomes of Myxococcota.

Myxococcota exhibit unique traits, encompassing motility [59], predation [60], fruiting bodies formation [61], and the ability to decompose cellulose [62] and catabolize aromatic lignin depolymerization products [63]. Here, we successfully assembled two abundant Myxococcota (Polyangiaceae) genomes expressing high cellulosic activity, including endoglucanase, exoglucanase, and cellobiohydrolase – enzymes exclusively found in Myxococcota MAGs. While Polyangiaceae also expressed CAZymes targeting bacterial biomass, the lack of chitinase prevented them from recycling fungal biomass (Fig. 3). Conversely, chitinases were consistently present in Bacteroidota and detected in Verrucomicrobiota, Pseudomonadota and Patescibacteria. Notably, although chitinase was found in only one copy in 5_UBA9983_A, this patescibacterial genome exhibited the second-highest chitinase expression.

Patescibacteria had been identified in a wide range of ecosystems [57, 64] including deadwood habitats [10, 65], yet their ecological role remains enigmatic. While their limited genome size, lacking crucial metabolic genes, suggests an obligate epibiotic lifestyle [66], their parasitic status remains subject to debate [67]. In our study, UBA9983_A demonstrated a possibility for specialized opportunistic lifestyle, primarily recycling chitin. Additionally, Patescibacteria of the order Saccharimonadales exhibited high expression of mannase, an enzyme involved in hemicellulose degradation, highlighting their active contribution in wood decomposition processes. These findings collectively imply that Patescibacteria may not solely depend on host resources. Further analyses are needed to elucidate whether hemicellulose-derived residues and chitin serve as energy sources for ATP generation or benefits to the host (e.g., commensalism or mutualism). Nevertheless, our results suggest a broader ecological relationship for Patescibacteria beyond parasitism.

Nitrogen concentration in deadwood is low [9] and represents a limiting factor for efficient decomposition. As metabolic pathways involved in nitrogen assimilation were scarce in the studied samples (Table S10), microbial biomass could represent an alternative source of nitrogen [68]. We found that CAZymes targeting bacterial biomass were more frequent than CAZymes targeting fungal biomass, but that peptidoglycanases were 2 times less expressed than chitinases (Table S4, Table S9). This result is surprising since fungal biomass contains less nitrogen than bacterial biomass [69], but these processes might simply be regulated by the availability of bacterial and eukaryotic biomass. While microbial biomass decomposition represents an interesting strategy for recycling nitrogen during decay, an external source of nitrogen is required to initiate decomposition of fresh dead wood. The initial nitrogen input is likely provided by bacterial nitrogen fixation, as illustrated by nitrogen fixation rates being eight times higher in young deadwood compared to old deadwood [9]. The conversion of atmospheric N_2_ to NH_3_, being a highly energy-consuming process [70], is only realized if no better suitable nitrogen sources are available [71]. The expression of nitrogenase genes at intermediate stages of decomposition (> 4 years) thus indicates that the recycling of microbial biomass does not completely meet the microbial nitrogen demand throughout the decomposition process.

Despite the fact that biological nitrogen fixation has been extensively investigated (e.g. [72, 73, 74]), our study marks the first observation of this function within the Steroidobacteraceae family. Beyond reporting a novel nitrogen-supplying bacteria in a nitrogen-limited environment, PacBio HiFi sequencing facilitated the investigation of genes involved in the conversion of N_2_ into ammonium. In the deadwood ecosystem, Pseudomonadota catalyze the production of reduced ferredoxin/flavodoxin using the fix operon, particularly advantageous under oxygen-limited conditions [75]. In addition, Steroidobacteraceae employ the *isc* system for biosynthesizing [Fe-S] proteins, crucial under elevated oxygen conditions [76]. The coexistence of the *fix* operon and the *isc* system likely allows Steroidobacteraceae to maintain nitrogen fixation activity under oxygen concentration fluctuations. Alphaproteobacteria appear less sensitive to elevated oxygen, favoring the *suf* system over the *isc* system, which is known to be more beneficial for bacterial growth in the presence of hydrogen peroxide [77]. Although the proportional contribution of Steroidobacteraceae (Gammaproteobacteria) and Alphaproteobacteria to global deadwood nitrogen fixation requires further exploration, our study provides empirical evidence of their in situ activity.

PacBio HiFi sequencing unveiled the remarkable potential of deadwood microorganisms for the production of diverse secondary metabolites, an important feature that would remain unnoticed in short-read data from this study. We identified over a thousand mostly novel BGCs, showcasing the extensive diversity of these BGCs in MAGs. This diversity suggests that deadwood bacteria display multiple interactions with other microorganisms in deadwood. Furthermore, our investigation highlighted bacterial groups in deadwood that hold promise for the production of novel bioactive compounds. Similar to observations in soil [78], the abundance of BGC types varied by taxonomy. In deadwood, we observed high numbers of BGCs in Myxococcota and Pseudomonadota, as well as in other groups such as Verrucomicrobiota and Acidobacteriota, whose biosynthetic potential has only recently been reported [79, 80]. Notably, we did not identify a shared BGC family across different bacterial taxa, indicative of a compound with broader importance in deadwood. In contrast, phylogenetic conservation of BGC families in deadwood MAGs was observed. The presence of BGCs for flexirubins, pigments typical of Bacteroidota [81], in several Chitinophagaceae MAGs constitutes convincing proof of validity of our metagenome analysis.

Long-read metagenomic analysis, while powerful, still has limitations. More effort is needed to appropriately describe the Planctomycetota: the high alpha diversity of this phylum in deadwood may require an increased sequencing depth for their genome recovery, as illustrated by the fact that Tláskal et al. [9] was able to recover one Planctomycetota MAG when combing short-read data from 25 deadwood samples (139 Gb) from the same forest. Nevertheless, long-read metagenomic sequencing provides substantial advantages in uncovering microbiome functions that are challenging to capture using short-read metagenomics. In our study, the short-read approach appeared reliable for identifying single-gene metabolic functions such as CAZymes, but failed to capture genomic features involving multiple genes, such as nitrogen fixation and biosynthetic gene clusters. Additionally, PacBio HiFi assemblies yielded more eukaryotic contigs than Illumina assemblies, although both platforms generated a proportional number of eukaryotic reads. This discrepancy is thus unlikely to stem from differences in the initial sample preparation for long-read sequencing (e.g., less intensive DNA extraction) but rather reflects the longer sequencing lengths and higher accuracy of PacBio HiFi sequencing [82]. Eukaryotic genomes, characterized by intricate features like repetitive regions, introns, and exons [83], require greater metagenomic sequencing depth and sample size to be successfully assembled compared to prokaryotic genomes. For instance, Saraiva et al., [84] needed 6000 terrestrial metagenomes to assemble 197 eukaryotic bins, whereas Ma et al., [85] achieved the assembly of 40,039 prokaryotic MAGs from 2941 soil metagenomes.

In conclusion, the PacBio HiFi sequencing enabled the assembly of both rare and abundant bacterial genomes, facilitating the assembly of novel genomes of bacteria with key roles in deadwood decomposition, such as cellulose decomposition in Polyangiaceae and nitrogen fixation in Steroidobacteraceae. Additionally, it revealed the significant contribution of Patescibacteria to wood decomposition processes and identified a wealth of new biosynthetic gene clusters with potential ecological and biotechnological significance.

## Supplementary Information

Supplementary material 1.

Supplementary material 2.

Supplementary material 3.

## Data Availability

Descriptions of the deadwood samples 6, 7, 57 and 84 are available at the NCBI BioSample repository (https://www.ncbi.nlm.nih.gov/biosample/), under accession numbers SAMN13925154, SAMN13925155, SAMN13925167, and SAMN13925168, respectively. The raw PacBio HiFi sequences from the four deadwood samples are available at the NCBI Sequence Read Archive repository (https://www.ncbi.nlm.nih.gov/sra/), under accession numbers SRR28211698—SRR28211701. The co-assembly of PacBio HiFi sequences is available at NCBI GenBank (https://www.ncbi.nlm.nih.gov/nuccore/) under accession number JBBCBH000000000. The 69 metagenome-assembled genomes from PacBio HiFi sequences are available at the NCBI GenBank repository, under accession numbers JBBCFX000000000—JBBCIN000000000. The raw Illumina HiSeq metagenome sequences corresponding to samples 6, 7, 57 and 84 are available at the NCBI Sequence Read Archive repository, under accession numbers SRR10968229, SRR10968228, SRR10968259, and SRR10968258, respectively. The Illumina HiSeq metatranscriptome sequences of samples 6 and 7 are available at the NCBI Sequence Read Archive repository, under accession numbers SRR10968251 and SRR10968250. The Illumina MiSeq 16S rRNA amplicon sequencing data for samples 6, 7, 57 and 84 are available at the NCBI Sequence Read Archive repository, under accession numbers SRR12914735, SRR12914734, SRR12914771, and SRR12914770. The biosynthetic gene clusters recovered from Illumina short-read metagenome, PacBio HiFi long-read metagenome and from PacBio HiFi MAGs, are available at the Zenodo repository (https://zenodo.org), under record numbers 10550953, 10529179, and 10529038, respectively.
