# Supplementary material for: Long-read sequencing sheds light on key bacteria contributing to deadwood decomposition processes
Source: Environ Microbiome. 2024 Dec 3;19:99. doi: 10.1186/s40793-024-00639-5 (PMC11613949; doi:10.1186/s40793-024-00639-5)
Supplement: Supplementary file 1 — Supplementary material 1. [file 40793_2024_639_MOESM1_ESM.docx]

**SUPPLEMENTARY MATERIAL**

**Pacbio HiFi sequencing sheds light on key bacteria contributing to deadwood decomposition processes**

Etienne Richy^1^*, Priscila Thiago Dobbler^1^, Vojtěch Tláskal^1,2^, Rubén López-Mondéjar^1,3^, Petr Baldrian^1^, Martina Kyselková^1^*

 1. Laboratory of Environmental Microbiology, Institute of Microbiology of the Czech Academy of Sciences, Vídeňská 1083, 14200 Prague 4, Czech Republic

2. Institute of Soil Biology and Biogeochemistry, Biology Centre of the Czech Academy of Sciences, Na Sádkách 7, 37005 České Budějovice, Czech Republic

3. Department of Soil and Water Conservation and Waste Management, CEBAS-CSIC, Campus Universitario de Espinardo, 30100, Murcia, Spain

*Corresponding authors: Martina Kyselková (martina.kyselkova@biomed.cas.cz) and Etienne Richy (etienne.richy@biomed.cas.cz)

## **1. Comparison of Illumina HiSeq and PacBio HiFi sequencing: taxonomic profiling, assembly, and binning**

**1.1. Material and Methods**

The metagenome and metatranscriptome of deadwood samples 6 and 7, previously sequenced with Illumina HiSeq 2500 (2 × 250 bases), were downloaded using the accession numbers SAMN13925154 and SAMN13762420, respectively (Tláskal et al. 2021). The metatranscriptomes of deadwood samples 57 and 84 being not available, only their metagenomes (Illumina HiSeq 2500, 2 × 250 bases) were downloaded using accession numbers SAMN13925167 and SAMN13925168, respectively (Tláskal et al. 2021).

The quality of the reads was assessed using Trimmomatic v0.36 (Bolger et al., 2014) and FASTX-Toolkit (http://hannonlab.cshl.edu/fastx_toolkit/), which removed adaptor contamination, low-quality reads (quality score <30), reads shorter than 50bp, and trimmed the low-quality ends of the reads. This resulted in a total of 84,675,162 sequences with a sum length of 19.8 Gb for the four metagenomes, and 83,442,180 sequences with a sum length of 8.6 Gb for the two metatranscriptomes (**Table S5**).

Illumina HiSeq raw reads were co-assembled and sample-by-sample assembled using megahit v1.2.9 (Li et al., 2015) with default settings. We also performed hybrid assemblies from short and long reads, using Unicycler v0.5.0 (Wick et al., 2017) with default settings. This was done on samples 57, 6 and 7 only, as the PacBio HiFi sequencing depth of sample 84 was too low (**Table S2**) and significantly increasing runtime.

Taxonomic profiling of the metagenome was performed using Kraken2 v2.1.2 (Wood et al., 2019) against the NCBI nt database (Wright et al., 2023) using default parameters. Binning of the short-read metagenomes was performed using the same approach as for the PacBio HiFi sequences; see the main text for details.

**1.2. Results and Discussion**

*1.2.1. Illumina HiSeq raw data and assemblies*

Illumina HiSeq sequencing generated an average of 21,168,790 sequences per sample with an average sum length of 4.9 Gb (**Table S5**). After quality filtering and adaptor trimming, the average sequence length was 234 bp. Fifty-one percent of Illumina raw reads were assigned to Prokaryotes (almost exclusively Bacteria), 19% were assigned to Eukaryotes and 30% remained unclassified (**Table S6**). While the percentage of eukaryotic raw reads was comparable between PacBio HiFi (long-read) and Illumina HiSeq (short-read) data, less prokaryotic raw reads were identified with Illumina, on the expense of unassigned reads.

The Illumina co-assembly produced 3,734,801 contigs, of which 102,279 contigs were greater than 1 kb and 109 were greater than 10 kb, with an N50 of 685 bp (**Table S1**). The largest contig had a size of 0.1 Mb, and the total length of assembled short-read contigs was around 0.6 Gb. The average proportion of short-read mapping to contigs was 39% (**Table S7**). Illumina contigs were mostly assigned to Bacteria, and 5.1% were assigned to Eukaryotes (**Table S6**), which is two-time lower than the number of eukaryotic contigs generated by Pacbio HiFi sequencing. See **Table S6** and **Table S2** for details on sample-by-sample assembly.

*1.2.2. Comparisons of short-read, long-read and hybrid assemblies*

Short-read and long-read sequencing approaches generated roughly similar amounts of data (an average of 4.9 Gb versus 4.0 Gb per sample respectively, **Table S5**), but PacBio HiFi sequencing produced more contiguous assemblies than Illumina. Notably, Hifiasm-meta produced the best assemblies for PacBio HiFi data, yielding twice the amount of data and longer contigs than Illumina megahit assemblies (**Table S1** and **Table S2**). Hybrid assembly of the data from both sequencing platforms combined was more computationally demanding and did not improve the number and size of contigs (**Table S2**). This is probably because hybrid assembly was originally developed to improve short-read assembly (Wick et al., 2017), as the sequencing error generated by long-read sequencing was high. However, the repetitive library which calls for consensus reads developed by the PacBio HiFi sequencing approach has significantly improved nucleotide accuracy (Marx, 2023). As a result, short reads seem to be no longer needed to improve the assembly of long reads if sequenced by PacBio HiFi approach. In our study, the hybrid assemblies produced contigs that were shorter in length than those generated by PacBio HiFi and fewer in number than those produced by Illumina (**Table S2**). Consequently, hybrid assembly was not further investigated.

*1.2.3. Comparisons of taxonomic profiles generated from short-read and long-read data*

The taxonomic profiles based on assembled short reads (A_SR) and assembled long reads (A_LR) were generally comparable (**Figure SM**), with the major taxonomic groups present in both short-read and long-read data. However, we observed differences in relative abundance of certain groups, which may result from different DNA extraction methods (less intense in the case of long reads to prevent shearing), library preparation, sequencing technology and the raw read length and quality. For example, Actinomycetota contigs were more abundant in A_SR, while A_LR contained higher share of fungal (Basidiomycota and Ascomycota) contigs. Contigs from Planctomycetota were consistently present across all methods. The reproducibility of long-read assemblies is further supported by the consistent taxonomic composition between raw reads (R_LR) and assembled reads (A_LR), indicating minimal bias during the assembly process. The only notable exception is sample 84, where the poor assembly profile likely reflects its low sequencing depth (19,734 sequences with an average sum length of 0.3 Gb, **Table S5**).


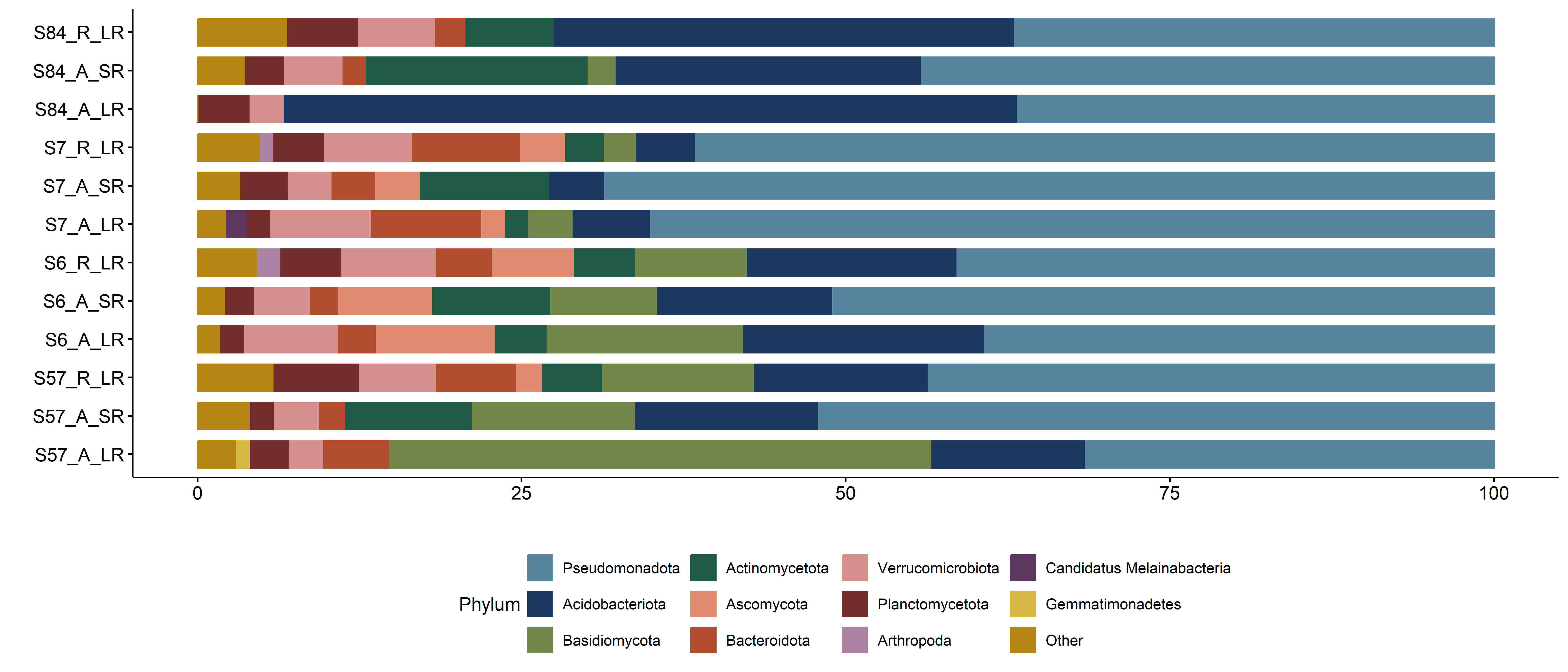


**Figure SM. Sample taxonomic profiling based on the assembled short reads (A_SR), assembled long reads (A_LR) and raw long reads (R_LR) using MEGAN-LR.**

*1.2.4. Illumina HiSeq data binning*

Eleven unique metagenome-assembled genomes (MAGs) were recovered from the Illumina HiSeq co-assembly and sample-by-sample assembly, all of which were Medium-quality draft MAGs (**Table S13**). These MAGs consisted of an average of 772 ± 603 contigs, with an average length of 3,480 ± 4,254 bp. Six of the 11 MAGs obtained from the short-read assemblies were also recovered from long-read data. In comparison to PacBio HiFi binning, Illumina HiSeq binning yielded six times less bacterial MAGs and the numbers of contigs per MAGs were on average 30 times higher (**Figure S1**). Consequently, these MAGs were not further analyzed.

## **2. Nitrogen fixation capability in Steroidobacteraceae**

**2.1. Material and Methods**

One of the nitrogen fixation contig (s509.ctg000513l) was found in the 36_Steroidobacterales MAG, a gammaproteobacterial genome from the family Steroidobacteraceae, assembled in this study. To explore nitrogen fixation potential in the Steroidobacteraceae family, we further analyzed 71 Steroidobacteraceae genomes from the GEMs catalog (https://portal.nersc.gov/GEM/) together with 110 isolated strain genomes of Steroidobacteraceae from NCBI. We annotated these genomes using eggnog-mapper v2.1.11, with the option ‘--itype metagenome’ for gene prediction from Diamond/MMseqs2 blastx hits, and no nitrogenase genes were identified (**Table S11**). Given the possibility of horizontal gene transfer (HGT) of the essential genes for nitrogen fixation (Bolhuis et al., 2010), we studied genomic islands of 36_Steroidobacterales with Islandviewer4 (Bertelli et al., 2017) after having identified the start position of the genome with circlator v1.5.5 (Hunt et al., 2015), and plotted the results using Proksee (Grant et al., 2023). We found no evidence of nitrogen fixation capability acquired from HGT in 36_Steroidobacterales. The genomic islands corresponded well to variation of GC content but occurred neither inside nor around the s509.ctg000513l contig (located between 5,118,742 and 5,335,241 bp) (**Figure 4c**).

We further examined the phylogenetic similarities of 36_Steroidobacterales *nifH* with sequences available in the InterPro database (accessed on August 1st, 2023). First, the NifH (amino acid) sequences were clustered at 97% of similarity using USEARCH v11.0.667. Then, multiple sequence alignment was generated using mafft v7.490 with the option ‘--maxiterate 1000’ and ‘--localpair’. We trimmed the alignment using BMGE v.2.0 software and the BLOSUM30 matrix. We computed the phylogenetic tree using iqtree v2.2.0.3 and the LG+C20 model and rooted the tree using minimal ancestor deviation (Tria et al., 2017). We next sub-selected the closest related taxa and used FigTree v1.4.4 for visualization. Finally, we confirmed the transcription of the nitrogen fixation genes in 36_Steroidobacterales by mapping the sample 7 transcripts (36_Steroidobacterales was assembled from this sample) to s509.ctg000513l contig using minimap2 v2.24 with ‘-x sr’ setting. Transcript counts were summed using dirseq v0.4.3 (Woodcroft et al., 2018) based on gene coordination from the gene prediction. We also confirmed the nitrogen fixation gene expression in the five PacBio HiFi contigs by mapping the sample 7 transcript to nitrogenase genes (*nifH*, *nifD*, *nifK*) (**Table S11**).

**2.2. Results and Discussion**

All nitrogenase genes identified in the five PacBio HiFi contigs were transcribed (**Figure 1A**), including those found in the 36_Steroidobacterales MAG (s509.ctg000513l). Transcript mapping against this MAG confirmed the expression of essential genes for nitrogen fixation (**Table S11**). The identification of nitrogen fixation genes within the Steroidobacteraceae family is noteworthy. Despite annotating 181 Steroidobacteraceae genomes from the Earth's Microbiomes catalog and the NCBI database (71 and 110 respectively, **Table S14**), revealing individual genes potentially implicated in nitrogen fixation (e.g., *fixB*, *iscASX*, *nifZ*, *sufBCES*, *fdx*), none of the annotated genomes encompassed the complete set of essential nitrogen fixation genes (**Table S11**). Moreover, extensive investigation of the NCBI nr and Interpro databases failed to identify *nifH* in the Steroidobacteraceae family (**Table S11**). Phylogenetic analyses of 36_Steroidobacterales *nifH* and the entire s509.ctg000513l contig indicated similarities with the Methyloccocales order (Gammaproteobacteria) (**Figure 4D**, **Table S11**), yet we found no evidence supporting recent HGT of this region into the 36_Steroidobacterales MAG (**Figure 4C**). Our analyses also found no support for mis-binning, affirming the accurate assembly of 36_Steroidobacterales (**Table S8**). Collectively, these data underscore the functional potential for nitrogen fixation within this deadwood-associated member of the Steroidobacteraceae family.

## **3. References**

Bertelli, C., Laird, M.R., Williams, K.P., Simon Fraser University Research Computing Group, Lau, B.Y., Hoad, G., Winsor, G.L., Brinkman, F.S., 2017. IslandViewer 4: expanded prediction of genomic islands for larger-scale datasets. Nucleic Acids Res. 45, W30–W35. https://doi.org/10.1093/nar/gkx343

Bolger, A.M., Lohse, M., Usadel, B., 2014. Trimmomatic: a flexible trimmer for Illumina sequence data. Bioinformatics 30, 2114–2120. https://doi.org/10.1093/bioinformatics/btu170

Bolhuis, H., Severin, I., Confurius-Guns, V., Wollenzien, U.I.A., Stal, L.J., 2010. Horizontal transfer of the nitrogen fixation gene cluster in the cyanobacterium *Microcoleus chthonoplastes*. ISME J. 4, 121–130. https://doi.org/10.1038/ismej.2009.99

Grant, J.R., Enns, E., Marinier, E., Mandal, A., Herman, E.K., Chen, C., Graham, M., Van Domselaar, G., Stothard, P., 2023. Proksee: in-depth characterization and visualization of bacterial genomes. Nucleic Acids Res. 51, W484–W492. https://doi.org/10.1093/nar/gkad326

Hunt, M., Silva, N.D., Otto, T.D., Parkhill, J., Keane, J.A., Harris, S.R., 2015. Circlator: automated circularization of genome assemblies using long sequencing reads. Genome Biol. 16, 294. https://doi.org/10.1186/s13059-015-0849-0

Marx, V., 2023. Method of the year: long-read sequencing. Nat. Methods 20, 6–11. https://doi.org/10.1038/s41592-022-01730-w

Tláskal, V., Brabcová, V., Větrovský, T., López-Mondéjar, R., Monteiro, L.M.O., Saraiva, J.P., Da Rocha, U.N., Baldrian, P., 2021. Metagenomes, metatranscriptomes and microbiomes of naturally decomposing deadwood. Sci. Data 8, 198. https://doi.org/10.1038/s41597-021-00987-8

Tria, F.D.K., Landan, G., Dagan, T., 2017. Phylogenetic rooting using minimal ancestor deviation. Nat. Ecol. Evol. 1, 0193. https://doi.org/10.1038/s41559-017-0193

Wick, R.R., Judd, L.M., Gorrie, C.L., Holt, K.E., 2017. Unicycler: Resolving bacterial genome assemblies from short and long sequencing reads. PLOS Comput. Biol. 13, e1005595. https://doi.org/10.1371/journal.pcbi.1005595

Wood, D.E., Lu, J., Langmead, B., 2019. Improved metagenomic analysis with Kraken 2. Genome Biol. 20, 257. https://doi.org/10.1186/s13059-019-1891-0

Woodcroft, B.J., Singleton, C.M., Boyd, J.A., Evans, P.N., Emerson, J.B., Zayed, A.A.F., Hoelzle, R.D., Lamberton, T.O., McCalley, C.K., Hodgkins, S.B., Wilson, R.M., Purvine, S.O., Nicora, C.D., Li, C., Frolking, S., Chanton, J.P., Crill, P.M., Saleska, S.R., Rich, V.I., Tyson, G.W., 2018. Genome-centric view of carbon processing in thawing permafrost. Nature 560, 49–54. https://doi.org/10.1038/s41586-018-0338-1

Wright, R.J., Comeau, A.M., Langille, M.G.I., 2023. From defaults to databases: parameter and database choice dramatically impact the performance of metagenomic taxonomic classification tools. Microb. Genomics 9. https://doi.org/10.1099/mgen.0.000949
