# Supplementary material for: Long-read sequencing sheds light on key bacteria contributing to deadwood decomposition processes
Source: Environ Microbiome. 2024 Dec 3;19:99. doi: 10.1186/s40793-024-00639-5 (PMC11613949; doi:10.1186/s40793-024-00639-5)
Supplement: Supplementary file 3 — Supplementary material 3. [file 40793_2024_639_MOESM3_ESM.pdf]

SUPPLEMENTARY FIGURES

Pacbio HiFi sequencing sheds light on key bacteria contributing to deadwood decomposition processes

Etienne Richy[1]*, Priscila Thiago Dobbler[1], Vojtěch Tláskal[1,2], Rubén López-Mondéjar[1,3], Petr Baldrian[1], Martina Kyselková[1]*

1. Laboratory of Environmental Microbiology, Institute of Microbiology of the Czech Academy of Sciences, Vídeňská 1083, 14200 Prague 4, Czech Republic

2. Institute of Soil Biology and Biogeochemistry, Biology Centre of the Czech Academy of Sciences, Na Sádkách 7, 37005 České Budějovice, Czech Republic

3. Department of Soil and Water Conservation and Waste Management, CEBAS-CSIC, Campus Universitario de Espinardo, 30100, Murcia, Spain

*Corresponding authors: Martina Kyselková (martina.kyselkova@biomed.cas.cz) and Etienne Richy (etienne.richy@biomed.cas.cz)

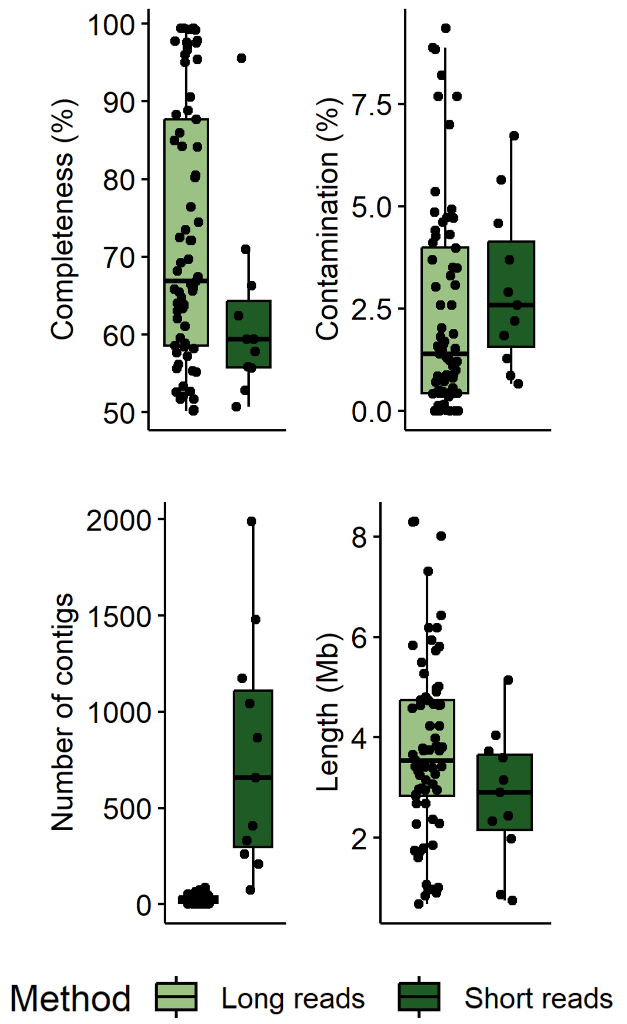

Figure S1. PacBio HiFi and Illumina binning statistics.

Genomic features of the unique MAGs generated from Pacbio HiFi assemblies (n = 69, light green) and Illumina Hiseq assemblies (n = 11, dark green). The completeness and the contamination were estimated using CheckM and the number of contigs and the size of the genomes (length) using seqkit tool.

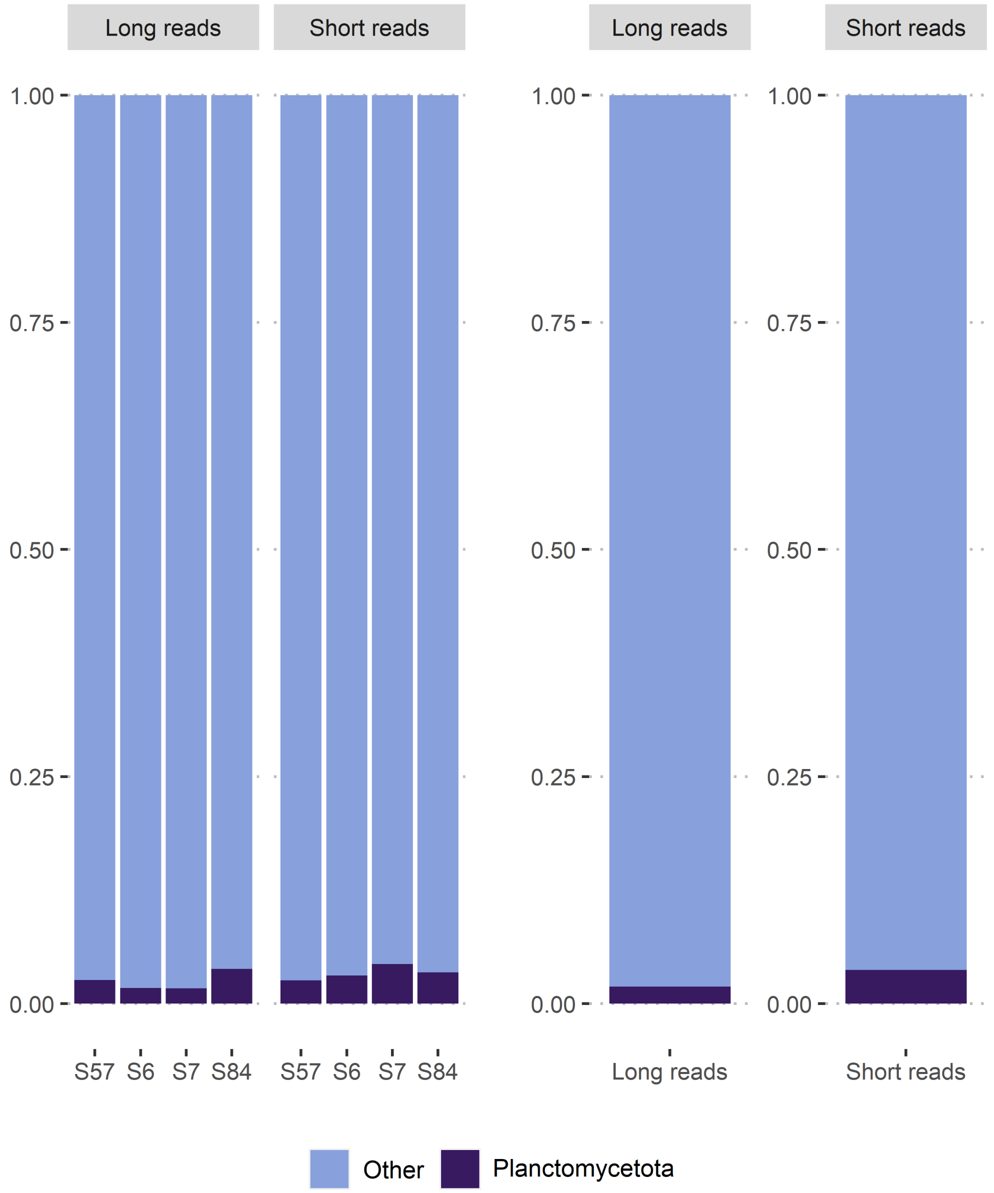

Figure S2. Proportion of Planctomycetota contigs.

Proportion of Planctomycetota contigs in sample-by-sample assembly and co-assembly of short and long reads. Assignment was based on the best blastp result against NCBI database nr.

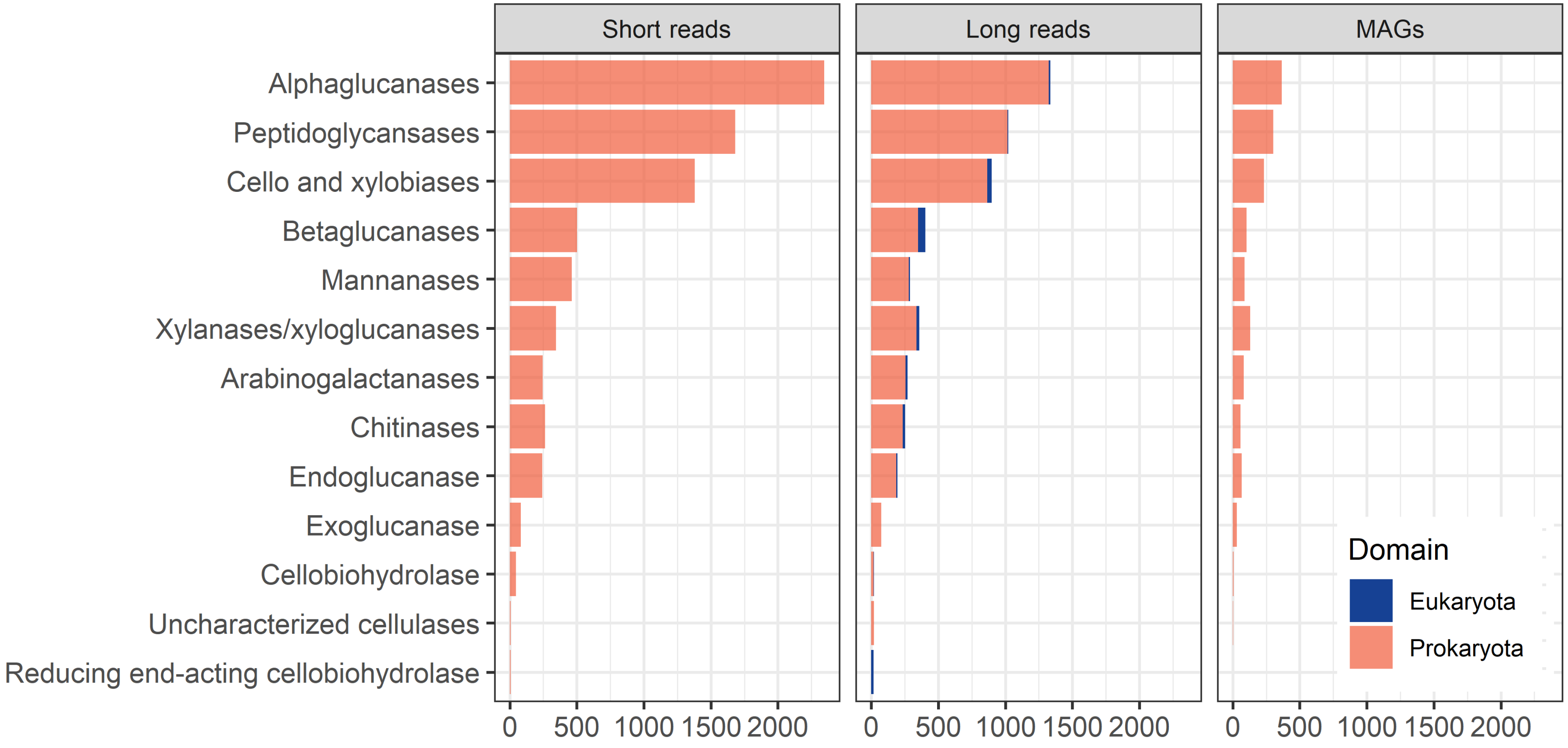

Figure S3. Carbohydrate active enzymes (CAZymes) identified in the short reads co-assembly, long reads co-assembly, and MAGs.

Numbers of CAZymes in short reads co-assembly (Illumina HiSeq), long reads co-assembly (PacBio HiFi), and MAGs (derived from PacBio HiFi assemblies). CAZymes are grouped by activity and ranked in descending order. Colours indicate the share of CAZyme families found in Eukaryotes and Prokaryotes. The majority of identified CAZymes were bacterial in origin. Short-read data did not yield any eukaryotic CAZymes, whereas 197 eukaryotic CAZymes were identified in the PacBio HiFi long reads data.

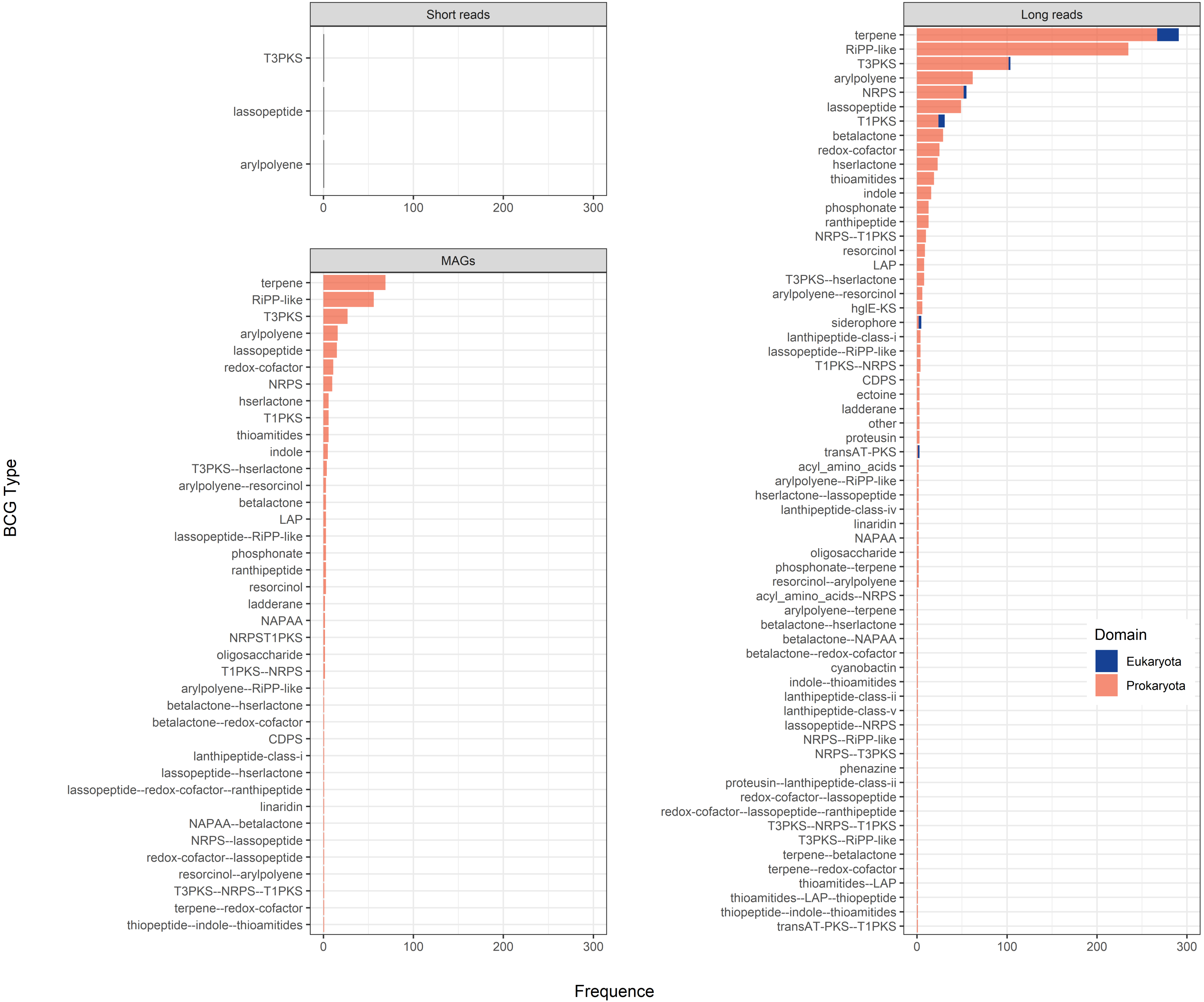

Figure S4. Biosynthetic gene clusters (BGCs) identified in the short reads co-assembly, long reads co-assembly, and MAGs.
Numbers of BGCs identified in short reads co-assembly (Illumina HiSeq), long reads co-assembly (PacBio HiFi), and MAGs (derived from PacBio HiFi assemblies). BGCs are categorized by type and ranked in descending order. Colors indicate the share of BGCs found in Eukaryotes and Prokaryotes. While the majority of BGCs were located on bacterial contigs, we also uncovered eukaryotic BGCs, including terpenes, non-ribosomally synthesized peptides (NRPS), polyketides, and siderophores. CDPS, tRNA-dependent cyclodipeptide synthase product; hserlactone, homoserine lactone; LAP, linear azol(in)e-containing peptides; NAPAA, non-alpha poly-amino acids like e-polylysin; NRPS, non-ribosomal peptide synthetase product; RiPP, ribosomally synthesised and post-translationally modified peptides; T1/T3 PKS, type I/type III polyketide synthase product.
